# Increased incidence of postoperative infections during prophylaxis with cephalothin compared to doxycycline in intestinal surgery

**DOI:** 10.1186/1471-2482-9-17

**Published:** 2009-12-07

**Authors:** Gunnar Baatrup, Roy M Nilsen, Rune Svensen, Per E Akselsen

**Affiliations:** 1 Department of Surgery, Haukeland University Hospital, Bergen, Norway; 2 Institute of Surgery, University of Bergen, Bergen, Norway; 3 Department of Infection Control, Haukeland University Hospital, Bergen, Norway; 4 Department of Public Health and Primary Health Care, University of Bergen, Bergen, Norway

## Abstract

**Background:**

The antibiotics used for prophylaxis during surgery may influence the rate of surgical site infections. Tetracyclines are attractive having a long half-life and few side effects when used in a single dose regimen. We studied the rate of surgical site infections during changing regimens of antibiotic prophylaxis in medium and major size surgery.

**Methods:**

Prospective registration of surgical site infection following intestinal resections and hysterectomies was performed. Possible confounding procedure and patient related factors were registered. The study included 1541 procedures and 1489 controls. The registration included time periods when the regimen was changed from doxycycline to cephalothin and back again.

**Results:**

The SSI in the colorectal department increased from 19% to 30% (p = 0.002) when doxycycline was substituted with cephalothin and decreased to 17% when we changed back to doxycycline (p = 0.005). In the gynaecology department the surgical site infection rate did not increase significantly. Subgroup analysis showed major changes in infections in rectal resections from 20% to 35% (p = 0.02) and back to 12% (p = 0.003).

**Conclusion:**

Doxycycline combined with metronidazole, is an attractive candidate for antibiotic prophylaxis in medium and major size intestinal surgery.

## Background

A high incidence of surgical site infections (SSIs) ranging from 11% to 26% after elective colorectal surgery has been reported by numerous authors during the last five years [1-5]. The incidence in unselected patient series, including urgent operations, patients with concurrent disease, patients in whom preoperative infections were suspected and patients with treatment failures is not known. Moderate size surgical procedures, such as small intestinal surgery and hysterectomies are followed by postoperative infection rates around 5% to 10%, or lower in selected elective cases [6-9]. About one half of the SSI becomes evident after discharge and the surgeons often do not know the true infection rate [1,10].

Internationally, cephalosporins dominate as the preferred group of drugs but several different regimens have been studied in attempts to reduce SSI [11-14]. Combinations of other β-lactam drugs, metronidazole and aminoglycosides are widely used alternatives and newer drugs, such as ertapenem, have also proved efficacious [3,15].

The decision on recommendations for antibiotic prophylaxis has to take many aspects into consideration. Factors like tissue penetration, mechanism of antibacterial action, half life in vivo, and protocol violation may be exchanged by the simple consideration: Does it work in the clinical everyday setting? It is important to consider the effect in routine settings because prospective, randomized trials will add focus on the topic, leading to a low degree of protocol violations and to results which might not reflect routine settings. Earlier reports have shown protocol violations in up to 40% [1,12,16]. Other considerations of importance are impact on bacterial resistance in the hospital and community, side effects, especially allergic reactions and induction of clinically important allergies.

As doxycycline is rarely used for therapeutic purposes in hospitals, it becomes more interesting as a prophylactic agent and it has been used in Norway for many years due to the early works of Giercksky *et al *[17]. It shows no cross allergy with more commonly used antibiotics, has a long half-life of 16 to 18 hours and a fair price. In preoperative prophylaxis one wants high antibacterial effect for a short period of time and doxycycline may have been overseen because it is bacteriostatic rather than bactericidal. Prophylactic drugs with shorter half-life demand strict regimens as to the timing of administration and drugs with long half-life are probably advantageous in single-dose regimens [18].

The aim of this study was to compare doxycycline and metronidazole with cephalothin and metronidazole as prophylactic agents against postoperative infections in an unselected population of medium and major size surgery. The analysis was performed in an everyday setting without focus on compliance of protocols of administration.

## Methods

The Department of Colorectal Surgery, and the Department of Gynaecology and Obstetrics, both Haukeland University Hospital, participated in the study. They are separate units with a common policy of preoperative antibiotic prophylaxis and with a common surveillance system of postoperative SSI. In the present study, we used surveillance data for the period January 1^st^, 2004 through February 29^th^, 2008. The Norwegian Social Science Data Services and the local ethics committee approved the study.

### Surveillance data

Surveillance of SSI after selected surgical procedures is mandatory in Norway with standardized collection of data. SSI for the NOMESCO codes JFB (resection of small or large intestine), JFH (colectomy), JFG (stoma and reservoir operations), JGB (resection of the rectum), LCD (excision of the uterus) and MCA 10 (elective, urgent or emergency caesarean section) were registered prospectively from January 1^st^, 2004.

A doctor registered SSI during hospital stay at discharge. All patients were mailed a questionnaire for infectious events 25-30 days after the operation. The patient questionnaire contained questions specific for signs of SSI and recommended the patient to see a doctor if such signs were evident. It also contained a questionnaire to the doctor who diagnosed the infection. The patients, who did not respond, received one reminder. Patients still hospitalized at the time of follow-up, were contacted and evaluated by Department of Infection Control for his/her infection status.

SSI was defined as surgical site infection within 30 days after the operation among those who were followed for 30 days. SSI was also categorized by degrees of severity as superficial incision, deep incision, or organ specific infection using CDC-definitions [19]. All out-of-hospital infections, except superficial SSI, had to be confirmed by a hospital department or a general practitioner. Patients experiencing SSI during the hospitalization and again after submission were registered as 2 separate events.

Patient related factors, such as age, gender and American Society of Anaesthesiologists (ASA) score and urgency were registered before the operation by the anesthesiologist. Procedure related factors, such as duration of the operation, and procedure code were registered in the operating theatre immediately after the operation. Emergency operations were those that had to be initiated within 2 hours from admission and urgent operations within 24 hours. Duration of the operation was registered as knife-time from incision to closure and as total time from entrance to the operating room to exit. Categorization of patients and procedure related variables are shown in Additional File 1. All surveillance data were registered electronically into databases managed by the Department of Infection Control.

### Antibiotics used for prophylaxis

Until July 31^st ^2006 the recommendation was 400 milligrams of doxycycline (Dumoxin^®^, Kipa Pharmacal Ltd.) combined with 1.5 grams of metronidazole for intestinal surgery and hysterectomies. By August 1^st ^2006, Dumoxin^® ^was withdrawn from the market due to manufacturing problems leading to loss of stability and reduced durability of the drug. We therefore changed the regimen to our second choice recommendation, which was 2.0 grams of the first generation cephalosporin cephalothin (Cefalotine^®^, ACS Dobfar Generics), combined with 1.5 grams of metronidazole. For surgical procedures lasting more than 3 hours, an additional dose of 2 grams of cephalothin was recommended. No antibiotic prophylactics were recommended for elective caesarean sections and 2 grams of cephalothin was recommended for emergency and urgent sections throughout the entire study period. The colorectal section registered an increase in SSI during the beginning of 2007, and several procedures related to SSI were scrutinized with no effect. By June 15^st ^2007, we therefore changed the prophylactic regimen to another commercially available doxycycline (Doxycycline^®^, Actavis). The department of Gynaecology and Obstetrics continued using cephalothin throughout the study period.

For the purpose of this study, a treatment variable with three time periods was coded according to the time period for which the various antibiotics were used (table 1). All preoperative antibiotics were administered intravenously. In the colorectal department the antibiotic infusion was initiated in the ward immediately before the patient was taken to the operation room and in the gynaecology department the infusion was initiated one hour before the patient was transferred to the operating theatre. The intestinal operations were initiated 55 minutes after they left the ward (mean time), except for rectal resections that had an epidural catheter before surgery and the operation started 75 minutes after they left the ward (mean time).

**Table 1:** Prophylactic antibiotic regimen according to patient group and period of observation

Patient group^a^	Period 1	Period 2	Period 3
Colorectal patients	Doxycycline and metronidazole	Cephalothin and metronidazole	Doxycycline and metronidazole
Gynaecology patients	Doxycycline and metronidazole	Cephalothin and metronidazole	Cephalothin and metronidazole
Patients with elective Caesarean section	None	None	None
Patients with acute Caesarean section	Cephalothin	Cephalothin	Cephalothin

^a ^The colorectal and the gynaecological patients are the intervention groups and the acute and elective caesarean sections are the control group.

### Patients

Initially, a total of 1882 procedures were performed and 1554 registrations (83%) were complete. Completeness of registration in the colorectal unit was 78% to 84% during the different periods. All resections of the small and large intestine were registered including rectal resections, but not appendectomies. In the gynaecology unit only hysterectomies were registered. The completeness of registration was 87% to 91%. If a patient was registered with the same surgical procedure more than once during the study period, only the first procedure was included in the study. This left us with a total of 1541 patients for analysis, 886 patients from the colorectal unit and 655 patients from the gynaecology unit (table 2). In addition, both elective and acute caesarean sections were included in the study as controls (1489 patients).

**Table 2:** Patient group and characteristics according to period of observation

Patient group and characteristics	Period 1	Period 2	Period 3
Colorectal patients (n)^a^	518	203	165
Patient's age (median)	68.0	70.0	65.0
Women (%)	49.0	46.8	49.7
ASA score (median)	2.0	2.0	2.0
Emergency procedure (%)	37.5	33.0	23.6
Day-time operation (%)	81.1	84.2	87.9
Operation time (median)	151	145	142
			
Gynaecology patients (n)^b^	365	153	137
Patient's age (median)	53.0	55.0	55.0
Women (%)	100	100	100
ASA score (median)	1.0	2.0	2.0
Emergency procedure (%)	1.64	1.31	1.46
Day-time operation (%)	97.5	100	99.3
Operation time (median)	100	117	115
			
Obstetric (control) patients (n)^c^	915	334	240
Patient's age (median)	31.0	31.0	31.0
Women (%)	100	100	100
ASA score (median)	1.0	2.0	2.0
Emergency procedure (%)	68.1	74.6	80.4
Day-time operation (%)	53.9	56.0	49.6
Operation time (median)	35.0	38.0	36.0

^a ^Colorectal procedures include all operations with resection of small or large intestine performed at the colorectal department.

^b ^Gynaecology procedures include excision of the uterus.

^c ^Obstetric procedures include acute and elective caesarean sections.

### Statistical analyses

All analyses were performed by using SAS (Statistical Analysis System) version 9.1 for windows (SAS Institute, Inc., Cary, North Carolina). All tests were two-sided and p-values below 0.05 were considered statistical significant. To examine if the incidence of SSI differed between various time periods with different recommendations for antibiotic prophylaxis, we used log-binomial regression analyses. Time periods were included in the models as a categorical variable, with cephalothin and metronidazole period as reference. Differences between the reference period and other time periods were measured by calculating crude and adjusted relative risks (RRs), with 95% confidence intervals (CIs). Adjustment variables were age, gender, surgical procedure, ASA score, urgency, time of operation, and operation time (Additional File 1). P-values were obtained from chi-square tests.

## Results

### Frequency of infections

**Figure 1:**
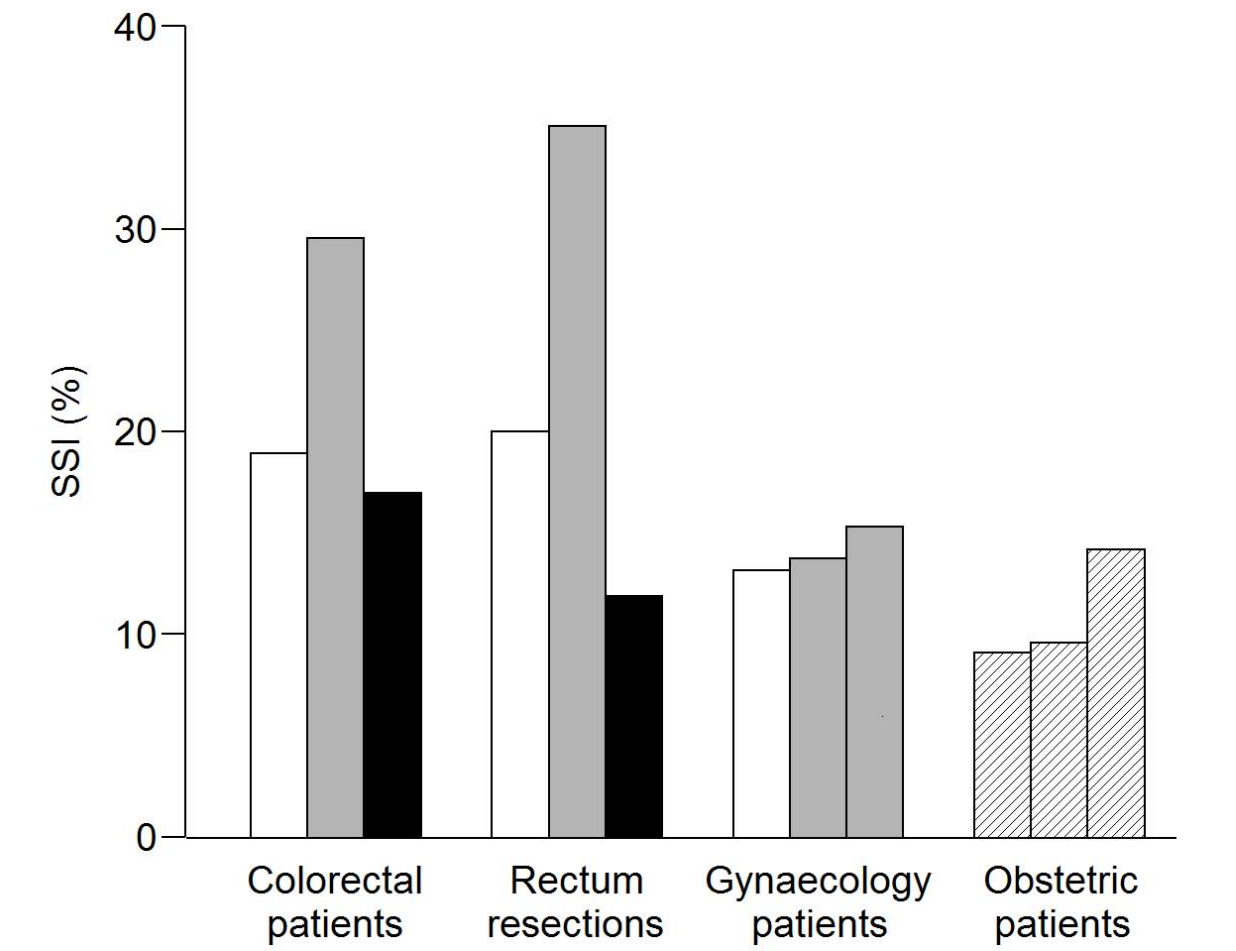
**SSI rates according to patient group and period of observation**. Period 1 was the first period of treatment with doxycycline and metronidazole (white bars). Period 2 was the period when cephalothin and metronidazole was used (grey bars). Period 3 was the period when we returned to doxycycline and metronidazole (black bars). The gynaecology department continued use of cephalothin and metronidazole during period 3 (i.e., same regimen during period 2 and 3). The obstetric (control) patients did not change the antibiotic regimen during the study period (i.e., hatched bars). Detailed data are provided in Additional File 2.

The period specific SSI rates for the various patient groups are given in figure 1. Period 1 is the 30 months prior to the shift of regimen from doxycycline and metronidazole to cephalothin and metronidazole. In the colorectal unit cephalothin was used during period 2 only, whereas it was used during period 2 and 3 in hysterectomies. The obstetric (control) patients did not change the antibiotic regimen during the study period.

For colorectal patients, the overall SSI rate increased from 18.9% to 29.6% (p = 0.002) during cephalothin prophylaxis as compared to doxycycline, and the high SSI rate was reversed to 17.0% (p = 0.005) when we returned to doxycycline in period 3 (figure 1). The SSI rate for gyneacology patients (i.e., hysterectomies) increased from 13.2% to 14.5% during the period of cephalothin prophylaxis (p = 0.28).

Both elective and acute caesarean sections were included into the registration (n = 1489). Elective caesarean sections received no antibiotic prophylactics in accordance with national recommendations and acute sections were recommended cephalothin only throughout the entire period of registration. The SSI rate in both elective and acute caesarean sections varied from 9.1% to 14.2% during the different periods of registration (figure 1).

Thirty-seven out of 63 rectal resections lasted more than 3 hours but only 27 of these patients received a second dose of antibiotics.

The number of patients who had deep incision or organ specific infections was too low for subgroup analysis (Additional File 2).

### Multivariate analysis

Table 3 shows crude and adjusted relative risks of SSI during the cephalothin and metronidazole periods compared to the doxycycline and metronidazole periods. There were no significant differences in infection rates between the time periods regarding hysterectomies (adjusted RR = 0.98, 95% CI: 0.64 - 1.49). A significant difference in SSI between the different time periods was found in all comparisons of colorectal procedures (table 3). In general, colorectal patients who had preoperative cephalothin and metronidazole were more likely to experience infections (overall adjusted RR = 1.61, 95% CI: 1.22 - 2.12), compared with those who had doxycycline and metronidazole. Adjustment for the variables listed in Additional File 1 had little or no impact on the effect estimates in any procedure group. Furthermore, of all potential confounders studied, only duration of the operation was significantly or borderline significantly correlated to the rate of SSI (p = 0.04 in hysterectomies and p = 0.05 in intestinal resections).

**Table 3:** Relative risks, with 95% confidence intervals, of SSI for periods with cephalothin and metronidazole relative to periods with doxycycline and metronidazole

Patient group	Periods	Crude RR^a^	95% CI	Adjusted RR^b^	95% CI
Colorectal patients^c^	2 versus 1 and 3	1.60	1.23, 2.09	1.61	1.22, 2.12
	2 versus 1	1.56	1.18, 2.06	1.57	1.18, 2.19
	2 versus 3	1.74	1.17, 2.60	1.74	1.15, 2.62
					
Gyneacology patients^d^	2 and 3 versus 1	1.10	0.75, 1.62	0.98	0.64, 1.49

^a ^Calculated by using log-binomial regression models.

^b ^Adjusted for age, gender, surgical procedure, ASA score, emergency procedure, time of operation and operation time.

^c ^Period 1 was the first period of treatment with doxycycline and metronidazole. Period 2 was the period when cephalothin and metronidazole was used, and period 3 was the period when we returned to doxycycline and metronidazole.

^d ^Period 1 was the first period of treatment with doxycycline and metronidazole. Cephalothin and metronidazole was used throughout period 2 and 3 (i.e., did not return to doxycycline and metronidazole in period 3).

### Subgroup analysis

In subgroup analyses of intestinal resections (figure 1), the rate of SSI was significantly higher during cephalothin prophylaxis in rectum resections (JGB procedures). The crude relative risk of SSI during period 2 (cephalothin) versus all other periods was 1.98 (95% CI: 1.25 - 3.12) and the adjusted RR was 2.19 (95% CI: 1.34 - 3.59). Infection rates were 20.0% and 11.9% during the two periods of doxycycline treatment (period 1 and 3) and 35.1% during cephalothin prophylaxis (period 2).

There was a trend towards a higher RR for SSI in surgery of the small and large intestine during the periods with cephalothin prophylaxis compared with the doxycycline periods (25.6% vs. 18.8% and 18.9%), but this was not significant (adjusted RR = 1.36, 95% CI: 0.95 - 1.93). The procedure groups "stoma and pouch procedures" (14 evaluated cases and 5 SSIs) and colectomies (54 evaluated cases and 11 SSIs) were too small for independent analysis.

## Discussion

This is a cohort study measuring the effect of changes in the recommendations for antibiotic prophylactics on an intention to treat basis. The results are convincing, as there is a good time relation between shift of recommendations for antibiotic prophylaxis and change in the SSI rate especially in the high-risk procedures like rectal resections. The results from the colorectal department are especially convincing, as shifts from doxycycline to cephalothin and back again were monitored. The SSI rate of caesarean sections increased non-significantly during the observation period. This further indicates that the observed changes in SSI for colorectal surgery may be related to the type of antibiotic used.

The infection rates during doxycycline prophylaxis are low compared with other published results taking into account that this is a non-selected patient population including all procedures on an intention to treat basis, not excluding high risk patients or cases with protocol violations. The number of patients is high and allowed precise effect estimates overall as well as in some subgroups.

There is an increase in SSI risk during cephalothin prophylaxis in intestinal surgery. The half-life of cephalothin is approximately 45 minutes compared to a half-life of 16-18 hrs for doxycycline. This short half-life renders patients receiving cephalothin prophylaxis vulnerable to protocol violations, which are known to be common and which happened in 27% of the cases [12,16]. Intravenous antibiotic prophylaxis should be administered 30-60 minutes prior to the operation. In the colorectal department patients received prophylaxis before they left the ward 55 minutes before the operation but 75 minutes before the operation for patients who had epidural catheter before surgery. The frequency of SSI could probably be reduced by reducing the number of protocol violations, and focus should be addressed to this. It is however well known that violations occur in the routine setting and it might be attractive to reduce this risk by using drugs with a longer half-life.

Duration of the procedure was the only factor amongst those monitored which was significantly related to the SSI rate. A significant increase in RR of SSI in long lasting procedures is well known [20]. It is therefore possible that strict adherence to recommendations of repeated administrations after 3 hours could reduce the SSI rate if cephalothin is used for prophylaxis. The 30-day SSI rate observed with cephalothin is, on the other hand, comparable to other rapports [1,3] and was consistently found to be reduced during doxycycline prophylaxis in 2 different departments with 2 different protocols for administration. Procedures for rectal surgery came out with the highest SSI. This is in accordance with the findings of Konishi *et al *[21].

It may seem surprising that the frequency of SSI during doxycycline-metronidazole prophylaxis is lower after rectal resections than after colon- and small intestinal resections. The frequency of acute procedures is, however low in the rectal resections and the level of experience of the principal surgeon higher. This cannot be verified by our data.

In the department of gynaecology, the difference in SSI during the 2 regimens is not significant.

## Conclusion

This study suggests that doxycycline in combination with metronidazole is a candidate that should be considered for prophylaxis in moderate and major size procedures in colorectal and small intestinal surgery and possibly in gynaecological procedures. It reduces the frequency of SSI in colorectal and small intestinal surgery and is comparable in effect with cephalothin in the other procedures. It is known as a safe drug causing few clinically important side effects.

## List of abbreviations

ASA: American Society of Anaesthesiologists; CI: Confidence interval; OR: Odds ratio; SSI: Surgical site infection.

## Competing interests

The authors declare that they have no competing interests.

## Authors' contributions

GB, RS, and PEA conceived the study and led the writing. RMN performed all statistical analyses. All authors helped to conceptualize ideas, interpret findings, and review drafts of the manuscript. No conflicts of interest are declared.

## Pre-publication history

The pre-publication history for this paper can be accessed here:

http://www.biomedcentral.com/1471-2482/9/17/prepub

## Supplementary Material

Additional file 1 **Categorization of patients and procedure related variables**. Table. Click here for file

Additional file 2 **SSI according to degree of severity, patient group and period of observation**. Table. Click here for file
