# Supplementary material for: Increased incidence of postoperative infections during prophylaxis with cephalothin compared to doxycycline in intestinal surgery
Source: BMC Surg. 2009 Dec 7;9:17. doi: 10.1186/1471-2482-9-17 (PMC2796642; doi:10.1186/1471-2482-9-17)
Supplement: Additional file 1 — Categorization of patients and procedure related variables. Table. [file 1471-2482-9-17-S1.DOC]

**Additional File 1:**

| Variables |  | Colorectal patients | | |  | Gynecology patients | | |
| --- | --- | --- | --- | --- | --- | --- | --- | --- |
|  |  | Subjects | SSI | |  | Subjects | SSI | |
|  |  |  |  |  |  |  |  |  |
|  |  | n | n | % |  | n | n | % |
| All patients |  | 886 | 186 | 21.0 |  | 655 | 90 | 13.7 |
|  |  |  |  |  |  |  |  |  |
| Age of the patient |  |  |  |  |  |  |  |  |
| Unknown |  | 1 | 0 | 0.0 |  | 0 | 0 |  |
| <25 years |  | 37 | 5 | 13.5 |  | 1 | 0 | 0.0 |
| 25-34 years |  | 22 | 5 | 22.7 |  | 22 | 2 | 9.1 |
| 35-44 years |  | 51 | 13 | 25.5 |  | 119 | 17 | 14.3 |
| >45 years |  | 775 | 163 | 21.0 |  | 513 | 71 | 13.8 |
| Gender of patient |  |  |  |  |  |  |  |  |
| Woman |  | 431 | 91 | 21.1 |  | 655 | 90 | 13.7 |
| Man |  | 455 | 95 | 20.9 |  |  |  |  |
| ASA score |  |  |  |  |  |  |  |  |
| Unknown |  | 24 | 4 | 16.7 |  | 24 | 5 | 20.8 |
| Healthy patient |  | 115 | 25 | 21.7 |  | 246 | 23 | 9.3 |
| Mild disease |  | 483 | 101 | 20.9 |  | 325 | 52 | 16.0 |
| Severe disease |  | 234 | 51 | 21.8 |  | 59 | 10 | 16.9 |
| Life-threatening disease |  | 29 | 5 | 17.2 |  | 1 | 0 | 0.0 |
| Moribund patient |  | 1 | 0 | 0.0 |  | 0 | 0 |  |
| Emergency procedure |  |  |  |  |  |  |  |  |
| Unknown |  | 0 | 0 |  |  | 4 | 0 | 0.0 |
| No |  | 586 | 122 | 20.8 |  | 641 | 89 | 13.9 |
| Yes |  | 300 | 64 | 21.3 |  | 10 | 1 | 10.0 |
| Time of operation |  |  |  |  |  |  |  |  |
| Unknown |  | 0 | 0 |  |  | 5 | 0 | 0.0 |
| Day (08.00-16.59 hours) |  | 736 | 160 | 21.7 |  | 645 | 89 | 13.8 |
| Evening (17.00-23.59 hours) |  | 95 | 18 | 18.9 |  | 3 | 1 | 33.3 |
| Night (00.00-07.59 hours) |  | 55 | 8 | 14.5 |  | 2 | 0 | 0.0 |
| Operation time |  |  |  |  |  |  |  |  |
| Unknown |  | 25 | 3 | 12.0 |  | 12 | 3 | 25.0 |
| 0-60 minutes |  | 19 | 3 | 15.8 |  | 54 | 2 | 3.7 |
| 61-120 minutes |  | 253 | 40 | 15.8 |  | 341 | 52 | 15.2 |
| 121-180 minutes |  | 316 | 71 | 22.5 |  | 179 | 27 | 15.1 |
| 181-240 minutes |  | 179 | 43 | 24.0 |  | 54 | 5 | 9.3 |
| 241-300 minutes |  | 64 | 18 | 28.1 |  | 12 | 1 | 8.3 |
| 301-360 minutes |  | 30 | 8 | 26.7 |  | 3 | 0 | 0.0 |
|  |  |  |  |  |  |  |  |  |
