# Supplementary material for: Increased incidence of postoperative infections during prophylaxis with cephalothin compared to doxycycline in intestinal surgery
Source: BMC Surg. 2009 Dec 7;9:17. doi: 10.1186/1471-2482-9-17 (PMC2796642; doi:10.1186/1471-2482-9-17)
Supplement: Additional file 2 — SSI according to degree of severity, patient group and period of observation. Table. [file 1471-2482-9-17-S2.DOC]

Additional File 2:

| Patient group |  | Period | Subjects |  | SSI | | | | | | | |
| --- | --- | --- | --- | --- | --- | --- | --- | --- | --- | --- | --- | --- |
|  |  |  |  |  | Any | | Superficial  incision | | Deep  incision | | Organ  specific | |
|  |  |  |  |  |  |  |  |  |  |  |  |  |
|  |  |  | n |  | n | % | n | % | n | % | n | % |
| Colorectal patients |  | All | 886 |  | 186 | 21.0 | 101 | 11.4 | 46 | 5.2 | 39 | 4.4 |
|  |  | 1^a^ | 518 |  | 98 | 18.9 | 54 | 10.4 | 24 | 4.6 | 20 | 3.9 |
|  |  | 2^b^ | 203 |  | 60 | 29.6 | 32 | 15.8 | 14 | 6.9 | 14 | 6.9 |
|  |  | 3^c^ | 165 |  | 28 | 17.0 | 15 | 9.1 | 8 | 4.8 | 5 | 3.0 |
|  |  |  |  |  |  |  |  |  |  |  |  |  |
| Gynaecology patients |  | All | 655 |  | 90 | 13.7 | 66 | 10.1 | 16 | 2.4 | 8 | 1.2 |
|  |  | 1^a^ | 365 |  | 48 | 13.2 | 32 | 8.8 | 10 | 2.7 | 6 | 1.6 |
|  |  | 2 and 3^d^ | 290 |  | 42 | 14.5 | 34 | 11.7 | 6 | 2.1 | 2 | 0.7 |
|  |  |  |  |  |  |  |  |  |  |  |  |  |
| Obstetric (control) patients |  | All^e^ | 1489 |  | 149 | 10.0 | 112 | 7.5 | 18 | 1.2 | 19 | 1.3 |
|  |  |  |  |  |  |  |  |  |  |  |  |  |

^a^ Period 1 was the first period of treatment with doxycycline and metronidazole.

^b^ Period 2 was the period when cephalothin and metronidazole was used.

^c^ Period 3 was the period when we returned to doxycycline and metronidazole.

^d^ The gynaecology department continued use of cephalothin and metronidazole during period 3 (i.e., same regimen during period 2 and 3).

**^e^** The obstetric (control) patients did not change the antibiotic regimen during the study period (i.e., same regimen during period 1, 2, and 3).
